# A practical approach to assess equity in fisheries management

**DOI:** 10.1038/s44183-026-00210-4

**Published:** 2026-06-03

**Authors:** Benjamin Dudouet, Olivier Thébaud, Arne Kinds, Sebastian Villasante

**Affiliations:** 1 EqualSea Lab – CRETUS, University of Santiago de Compostela, Santiago de Compostela, Spain; 2 Ifremer, Univ Brest, CNRS, IRD, UMR 6308 AMURE, Unité d’Economie Maritime, IUEM, Plouzané, France; 3 Oportunius Distinguished Researcher Professor, Xunta de Galicia, Santiago de compostela, Spain

**Keywords:** Ecology, Ecology, Environmental sciences, Environmental social sciences, Ocean sciences

## Abstract

A corner-stone of fisheries management is the regulation of access to the fisheries stocks through the allocation of fishing opportunities which aim to limit the “race-to-fish”, leading to increased economic efficiency. It is widely recognized that the equitable distribution of fisheries access rights is key to the acceptability and effectiveness of such regulatory systems. This dimension, however, remains largely underexplored. We develop a practical approach to assess equity in fisheries management systems. Building on the conceptual foundations of the fisheries social contract, we define equity through a three-tiered framework and propose a scoring system for its evaluation. We empirically test the approach through a comparative analysis of the French and Spanish allocation regimes for the Bay of Biscay Hake (*Merluccius merluccius*) fishery. The findings highlight the importance of explicitly incorporating equity into fisheries governance alongside ecological and economic objectives.

## Introduction

Sustainable fisheries management extends beyond setting target levels of fishing mortality and harvest, such as Maximum Sustainable Yield (MSY), through the use of management measures such as Total Allowable Catches (TAC)^1–3^. Indeed, given that total catch limits implementation can ultimately lead to a “race to fish”, creating economic inefficiencies and negative social outcomes. Another cornerstone of fisheries management is to regulate access to the fisheries stocks through the allocation of individual fishing opportunities. Access to fish stocks can be regulated through a range of alternative approaches that vary according to the control variable (effort or catch) and the method of allocation, leading to a diversity of institutional arrangements^4,5^. By aligning individual incentives with collective sustainability goals, these measures are expected to help increase economic efficiency and support for the collective harvesting targets^6,7^.

Numerous studies that have adapted and applied a wide range of methods to evaluate the economic, ecological and social impacts of access regulation systems in fisheries worldwide ^8–16^. The development of evaluation frameworks addressing these social dimensions, and in particular equity, remains widely underexplored. This may be because, while equity is broadly defined as the fair and just distribution of resources, its application to fisheries management is far from straightforward. The creation, allocation, and utilization of fishing rights raise ethical concerns pertinent to present and future human societies^17^. Indeed, as they involve excluding certain operators and deciding on who can fish and how much, these rules raise important equity questions^18–21^. How these questions are addressed may, in turn, determine the support for the fisheries management systems, extending to the entire set of management rules. Jentoft^22^ argues that when rules are perceived as legitimate, they encourage consent and collaboration among stakeholders, which is essential for effective fisheries management. Equity has gained traction as a central theme in marine governance^10,23,24^, and is frequently cited as a key principle to increase legitimacy and consent in decision-making and governance processes^25–29^. Concerns about equity in the allocation of access rights within fisheries management systems have also increasingly been addressed in the scientific literature. Scholars have examined various dimensions of rights distribution, including track-record-based allocation^30^ and concerns about quota leasing^31^. Several studies have questioned whether equity is meaningfully integrated into income distributional mechanisms^32,33^, while others have emphasized the need to make equity a central management objective in individual transferable quota (ITQ) systems^34,35^. Legal analyzes have also explored how equity is considered within rights allocation^16^, including its implications for future fisher generations^19^. Rather than focusing solely on formal rights, Kourantidou et al.^20^ argue for a broader integration of equity into fisheries governance by acknowledging the specific claims of local and Indigenous Peoples. Additional contributions have highlighted the importance of legitimacy^22^ and the social contract in quota-based regimes^36,37^. Recent work has also highlighted the inherent trade-offs between equity and efficiency in fisheries policy design, emphasizing the need to recognize equity not only as a constraint but as a legitimate and explicit objective^38,39^ or suggesting using social criteria to address outcomes and opportunities inequalities^33^. Campbell and Hanich^40^ offer a comprehensive conceptualization of equity linking it to the distribution of rights, responsibilities, and the associated costs and benefits, providing a useful starting point for applying equity consideration in fisheries.

Despite these advances and the rich, multidimensional discussion in the literature, a coherent and systematic framework for assessing equity of fisheries management systems - comparable to those used for evaluating their ecological and economic performance - remains largely absent, representing an important gap in current research. A major challenge in evaluating these systems lies in the lack of a clear and operational definition of equity

This study develops a new approach to operationalize and assess the equity of regulatory systems, determining the allocation of fishing access rights. It involves a stepwise approach, built on a conceptual three-tiered framework (sensu “decision making”, “management” and “distribution”), for analyzing and comparing fisheries management systems as they relate to the allocation of fishing possibilities, from an equity perspective.

We base our approach on the concept of a fishery social contract, which offers a valuable lens through which to operationalize equity assessment of an organized fisheries management system. We consider that such a fishery social contract sets the rules that balance resource use with collective well-being and shape expectations of fairness, responsibility, and legitimacy^17,41^. While the social contract underpinning allocation systems shares a common foundational principle, its specific manifestations may vary significantly across countries and contexts, reflecting divergent priorities of fishery stakeholders (e.g., a focus on large-scale production, community-based access, or state-controlled allocation)^42,43^.

Based on our definition of a fishery social contract, we develop a three-tiered framework to assess the equity in a fisheries management context. To apply this framework, we develop a scoring system composed of 31 questions to empirically evaluate different dimensions of equity. We then apply this scoring system to the case of hake (*Merluccius merluccius*) fisheries in the Bay of Biscay, with a specific focus on comparing the French and Spanish systems.

Hake is a species of significant economic importance in France and Spain, representing a single stock, managed under the same European Common Fisheries Policy (CFP) as regards the determination of conservation targets, but with each country adopting its own allocation regime for the resulting fishing possibilities^44,45^. Since the allocation of fishing opportunities is delegated to the EU member states, two markedly different management systems have developed: The Spanish fisheries management system, placing greater emphasis on centralized regulation, and the French, more decentralized system. This social-ecological contrast provides an ideal setting to test our approach and assess its potential for policymakers to compare the two management systems.

## Results

This section presents both the conceptual framework developed to conceptualize equity in fisheries management and the practical assessment derived from an empirical application, aimed at demonstrating the practical applicability of this approach in assessing equity across specific contexts.

### A three-tiered framework to conceptualize equitable fisheries

**Fig. 1:**
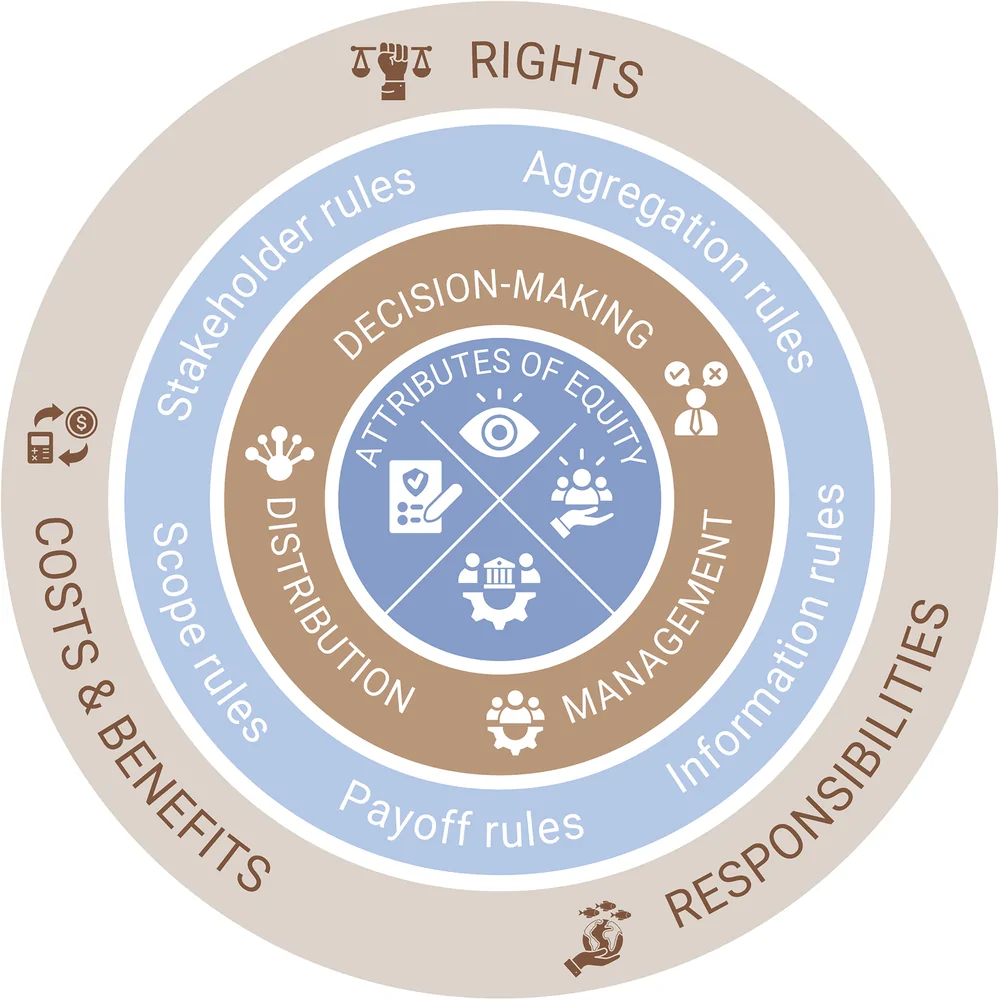
Conceptual framework of the fisheries access regulation systems (own realisation in collaboration with “Azuli Design”). It presents a schematic representation of the key components of an equitable fisheries management system, organized into four concentric circles radiating from the center. The innermost circle outlines the four attributes of equity. The second circle depicts the three core dimensions of fisheries management. The third circle identifies the five types of rules that structure management systems. Finally, the outermost circle illustrates the rights, responsibilities, costs and benefits distributed by the system.

In order to operationalize equity within the context of fisheries management, we develop a conceptual framework (Fig. 1) built upon both scientific literature^40,46–51^ and expert knowledge of co-authors.

Figure 1 illustrates the attributes of equity as they apply to the three components involved in setting and applying rules for the establishment and distribution of rights, responsibilities, costs and benefits. The figure should be read from the center of the circle outward, illustrating how rights, costs, benefits and responsibilities (fourth circle) are distributed and applied at each stage of the process (second circle) by the different rules (third circle).

Based on the literature review, we propose a set of attributes of equity in fisheries management: clarity, clarity of the responsibility, legitimacy, and mechanisms and institutions supporting management costs^22,40,41,50,52,53^. The center part of Fig. 1 summarizes these critical elements, highlighting clarity of definition and responsibilities, legitimacy and the presence of mechanisms to support management costs.

First, “clarity” is a foundational attribute of any just system (Top-central symbol of Fig. 1), as it ensures that all stakeholders share a common understanding of the rules of their social contract^41^. A clear definition of rules-is-use allows for both informed participation in the social contract and consistent application of its norms^52,54^.

Second, the “clarity of responsibility” establishes well-defined roles and duties for each actor (right-central symbol of Fig. 1). Responsibilities refer to the obligations tied to specific roles, rights, or actions. As Sen^53^ argues, freedom and capability encompass not only opportunities but also responsibility for the use of those rights, ensuring that actors are held responsible for fulfilling the obligations that accompany their use of the rights. In the context of natural resource management, Campbell and Hanich^40^ emphasize the clarity of responsibility as a key marker for equitable management. By defining responsibilities clearly, the system promotes fairness, ensuring that each participant is held to standards appropriate to their role.

Third, the “legitimacy” of the rules ensures that regulations are perceived as just, fostering consent and trust among stakeholders (Left-central symbol of Fig. 1). According to Jentoft^22^, when rules are viewed as legitimate, they foster consent and cooperation among actors involved.

Finally, the presence of “mechanisms and institutions supporting management costs” guarantees that the financial and administrative burden of management is viable^55^ (symbol at the lower-center of Fig. 1). In the context of natural resource management, especially under co-management arrangements, transaction costs can be substantial^50^. Equitable management thus requires not only the design of fair rules and roles, but also the capacity to implement them without overburdening any particular stakeholder group, and accounting for preferential rights of less advantaged stakeholders.

To evaluate equity, we structure our analysis around 3 components that reflect the temporal^46^ and institutional dimensions^48^ of fisheries management as follows:

1. *Decision making:* The formulation of the social contract and institutional environment. The long-term decision-making processes that establish strategic orientations. For example, this relates to international and national long-term and structural rules.
2. *Management:* The implementation of the social contract, medium-term management decisions and institutional arrangements, such as annual or seasonal rules. For instance, this relates to the national annual access rights allocation.
3. *Distribution:* The distribution of access rights, short-term decisions related to resource allocation, such as the daily monitoring and reallocation of access rights. For example, this relates to the daily access rights reallocation operated by the diverse institutions.

Each of these components operates within a hierarchy of interrelated rules. In this respect, the notion of rules-in-use offers a powerful analytical tool to unpack the inner workings of the social contract in fisheries management^56^. These rules offer a structured lens for analyzing how decisions are made, who participates, what actions are permitted, and how benefits and responsibilities are distributed across management levels. Applied to access regulation systems, rules-in-use describe the architecture of the social contract. Each rule defines a different aspect of management. This approach enables a fine-grained analysis of how equity is embedded within institutional design. The rules, inspired from the Ostrom et al.^56^ rules-in-use, are the following (Fig. 1): Stakeholders rules (about the stakeholders' roles), Aggregation rules (about the decision process), Information rules (the information availability), Payoff rules (the rights distribution) and the scope rules (the objective of the system) (Fig. 1).

To assess equity in practice, it is essential to examine the rules of each step of the framework (decision-making, management and distribution) through the lens of rights, responsibilities, and costs and benefits^40^. These components serve not only as diagnostic criteria, but also as normative principles that guide the design and evaluation of equitable institutional arrangements.

Rights define what individuals or groups are legally or morally entitled to do or to receive. In the context of fisheries management, this includes both access rights (the legal ability to enter a fishery) and harvest rights (the right to extract a share of the resource)^57^. These entitlements—whether formal or customary—are critical in determining who participates in, and who benefits from, fisheries systems.

Responsibilities refer to the duties or obligations associated with specific roles, rights, or actions. As Sen^53^ argues, freedom and capability imply not only opportunities but also accountability. In fisheries management, this means that actors who hold decision-making power or resource access must be accountable for their actions and the outcomes they produce, particularly regarding the sustainability of fish stocks, equitable distribution of access rights and the well-being of fishing communities^17^.

Finally, the distribution of costs and benefits lies at the core of distributive justice. Articulated by Rawls^52^ and Deutsch^58^, justice entails fair sharing of the burdens and benefits generated by collective action. In fisheries management, these costs and benefits may be material (e.g., income, access to fish stocks, compliance costs) or intangible (e.g., political voice, cultural recognition).

Among these, transaction costs represent a crucial yet often overlooked component of the overall distribution. They encompass the costs arising from institutional arrangements and social interactions^59^, including implementation, coordination, and enforcement of rules within the fisheries social contract. McCann et al.^60^ propose a framework for categorizing and measuring such costs in environmental policy contexts, while Marshall^50^ demonstrates their influence on institutional choice and collective action. Viewed through Ostrom’s rules-in-use^61^, transaction costs thus highlight the economic dimension of equity, revealing how management arrangements may impose differing burdens on actors.

### The equity assessment approach

This section outlines the assessment approach used to evaluate equity in fisheries access rights allocation. It presents the key attributes of equity and introduces the questionnaires developed to measure them systematically, based on an established three-tiered framework.

We develop a scoring system based on the above equity dimensions to assess the allocation mechanisms in practical situations. This grading system of 31 questions evaluates rights, responsibilities, costs and benefits to assess the overall equity of a fishery management system. Each category includes a specific question and a ten-level scoring scale to measure the quality or effectiveness of the criteria being evaluated (Supplementary Table 3). This scale provides sufficient granularity to capture subtle differences while remaining intuitive for respondents and also facilitates statistical analysis. The scoring system is applicable to the three-tiered framework of the fisheries access regulation system (“decision making”, “management” and “distribution”).

The scores about “Rights” and “Responsibilities” ensure that all stakeholders have a common understanding of their roles and obligations, fostering accountability and transparency. The “costs” part evaluates whether the financial and operational burdens incurred are manageable within the framework provided. Finally, “benefits” category guarantees that the advantages of the system are legitimate and promote equity. The scoring system for each of these levels is further described hereafter.

This first step focuses exclusively on the general context of the management system, which is evaluated through the first part of the scoring system designed to assess the interannual management framework of a fishery (Table 1).

**Table 1:** “Decision-making” component of the scoring system (Own realisation)

Stakeholders rules	Are the roles of each stakeholder in the decision process explicit and clear?
Aggregation rules	Is the pluri-annual decision-making process clearly defined?
	Is the long-term decision process legitimate?
	Are there institutions or mechanisms in place to support the decision-making process (help with access to information, public participation, discussion/debate forums)?
Information rules	Is the pluri-annual information access clearly defined?
	Is it clearly defined who is responsible to give access to the information?
	Is the pluri-annual information equally accessible for all the stakeholders?
	Are there any institutions or mechanisms in place to support the distribution of clear information?
Payoff rules	Are the pluri-annual rules to distribute the fishing possibilities clearly defined?
	Are the pluri-annual distribution rules of fishing possibilities legitimate?
Scope rules	Is the purpose of the pluri-annual fishery management clearly defined?
	Is the purpose of the fisheries policies legitimate?

First, *stakeholder rules* concern the explicit definition of roles and responsibilities in the decision-making process, ensuring that each actor understands their position and influence. *Aggregation rules* then evaluate whether the long-term decision-making process is formally established, perceived as legitimate, and supported by mechanisms that support transaction costs.

*Information rules* assess whether rights of access to information are clearly defined, whether responsibilities for providing information are explicitly defined, equally available and if mechanisms or institutions support the information distribution. *Payoff rules* focus on the distribution of fisheries access rights, examining whether allocation rules are not only clearly formulated but also recognized as legitimate. The legitimacy of pluri-annual distribution rules for fishing opportunities reflects the recognition of stakeholders as legitimate participants within the frameworks established by each country’s social contract.

Finally, *scope rules* assess whether the purpose of a long-term decision is clearly defined and considered as legitimate.

This second step focuses exclusively on the mid-term management system, which is evaluated through the second part of the scoring system designed to assess the annual or seasonal management framework of a given fishery (Table 2).

**Table 2:** “Management” component of the scoring system (Own realisation)

Stakeholders rules	Are the roles of each stakeholder in the seasonal fisheries management explicit and clear?
Aggregation rules	Is the process to decide the seasonal management strategy clearly defined?
	Is the way of deciding the seasonal management strategy legitimate?
	Are there institutions or mechanisms in place to support the seasonal decision-making process?
Information rules	Is it clear who gets the right to access the information at the beginning of the season to edit the fisheries management strategy?
	Is it clearly defined who is responsible to provide the information at the beginning of the season to be able to take the seasonal decision?
	Is the information equally accessible for all the stakeholders?
	Are there any institutions or mechanisms in place to support the distribution of clear information?
Payoff rules	Is it clearly defined who receives the fishing rights in the seasonal fisheries management strategy?
	Are the annual distribution rules of fishing possibilities legitimate?
	Are the costs associated with the seasonal distribution of the fishing rights supportable?
Scope rules	Is the purpose of the seasonal management strategy and the actors responsible for it clearly defined?

*Stakeholders rules* questions examine whether the roles and responsibilities of different actors in the seasonal management process are explicitly defined. *Aggregation rules* address the organization of the seasonal decision-making process, evaluating whether the definition of the process, its legitimacy and presence of mechanisms and institutions supporting the decision process costs.

*Information rules* assess whether the establishment of the rights to access the information, the assignment of the responsibilities for providing such information, the equality of access and finally consider the existence of mechanisms that ensure the distribution.

*Payoff rules* examine whether the rules governing the distribution of fishing rights are clear, whether they are legitimate and whether the costs associated with their implementation are bearable. Finally, the *scope rules* question evaluates whether the objectives and responsible actors are clearly identified.

The last part of the scoring system evaluates the infra-annual fisheries management system, focusing on short-term management (Table 3).

**Table 3:** “Distribution” component of the scoring system (Own realisation)

Stakeholders rules	Are the rights and responsibilities well defined in the infra-annual fisheries management system?
	Is the previous repartition of the daily rights and responsibilities between stakeholders legitimate?
Aggregation rules	Does it clearly define how decisions are made during the fishing season?
Information rules	Is it clearly defined who gets the rights to access the infra-annual information to manage the fishery? And who is responsible to give this access?
	Is daily information accessible in the same way to all stakeholders?
Scope rules	Is the purpose of the infra-annual management clear?
**Costs**	Are there institutions and mechanisms supporting the intra-annual management of the fishery?

*Stakeholders rules* assess whether rights and responsibilities within the infra-annual system are clearly defined, and whether the previous distribution is considered legitimate. *Aggregation rules* then examine whether decision-making procedures during fishing seasons are explicitly established.

*Information rules* assess the clarity of the definition of who has the right to access the daily information, and who has the responsibility to provide it, and whether such information is equally available to all stakeholders. *Scope rules* further consider whether the purpose of infra-annual management is clearly defined. The final question involves assessing whether appropriate institutions and mechanisms exist to support the daily or short-term management of the fishery.

### Empirical application

We demonstrate the applicability of this approach by applying it to a case study: the Hake fishery in the Bay of Biscay, which spans both France and Spain, thus providing an informative test case. The fishery offers valuable insights into the challenges and successes of managing shared marine resources, highlighting the complexities of the distribution of fishing rights and responsibilities between stakeholders in different national contexts.

France and Spain manage their hake fisheries in the Bay of Biscay under the framework of the CFP, which ensures sustainability and stock conservation through quota systems, technical measures, and monitoring requirements^1,2^. This allows for a uniform implementation of CFP rules, providing clearer regulatory pathways for fishers and stakeholders. Every year, both countries receive part of the annually decided TAC according to the relative stability (Le Floc’h et al.^1^, Marchal et al.^2^). In 2022, Spain received 13,984 tons of Hake (8ABDE + 8C3411) and France 20,871 tons of Hake (8ABDE + 8C3411). Then, annually, the quotas are spread directly or indirectly to the fishing vessels as fishing possibilities.

The European hake fisheries (*Merluccius merluccius*) in the Bay of Biscay are important for both France and Spain. In the Southern Western water, mainly operated by France, Spain and Portugal, European Hake is the main species in value and ref. ^62^. France and Spain landed 94% of the European hake value in 2022.

While both France and Spain follow the multi-annual frameworks set by the CFP, annual decision-making allows for greater national discretion, revealing differences in management structures. Despite these structural differences, in both countries, decision-making is increasingly supported by co-management institutions, which play a crucial role in facilitating collective management^15,46,55^.

Thus, both Spain and France operate systems where fishing quotas are largely allocated on the basis of individual entitlements, but are subsequently managed and collectively optimized by co-management organizations, thereby enabling a partial pooling of fishing opportunities while maintaining the framework of individual allocations (Table 4). While Spain and France both have POs as key institutions in fisheries management, their degree of authority and operational influence differ.

**Table 4:** French and Spanish Bay of Biscay Hake fisheries description

France	Spain
In France, the shares of TAC are allocated directly to POs, according to their members’ track records, which then assume primary responsibility for distributing quotas among their members. POs receive the quota allocations directly and are thus deeply embedded in decision-making processes, with formalized powers to allocate and manage quotas directly. The French system thus empowers POs to play a key role in both the management of quotas.	The shares of the TAC allocated to Spain are first distributed by the central administration to individual vessels, based on historical catch records and vessel characteristics. However, once allocated, co-management institutions (e.g., POs, vessel owners’ associations and Cofradias) retain the capacity to internally redistribute these quotas among their members. This process allows for a degree of operational adaptation capacity. The POs facilitate the internal redistribution of the individual entitlements held by their members.

Through this case study, we demonstrate the usefulness of our approach in assessing the equity of fisheries management systems. The results are derived from the authors’ expert knowledge. As such, they are only exploratory and intended for illustrative purposes.

**Fig. 2:**
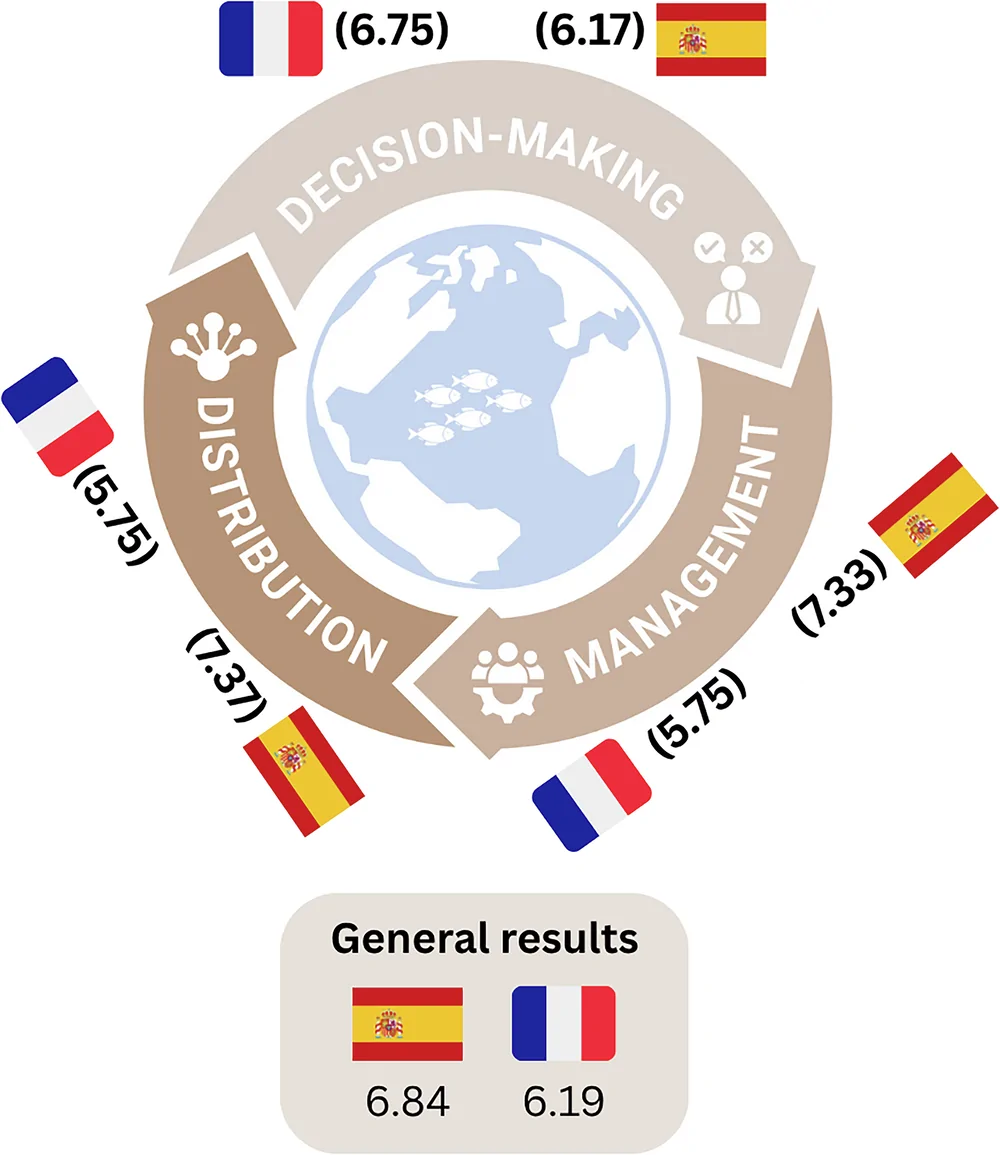
Results of the scoring system for the hake fishery in France and Spain (Own realisation). Building on Fig. 1, this figure overlays empirical findings from case studies of the French and Spanish hake fisheries in the Bay of Baiscay. Results for each country are distinguished by their respective national flags.

The average equity scores reveal differences between the two countries. Overall, Spain attains a slightly higher mean score (6.84) compared to France (6.19), indicating a generally more equitable management system (Fig. 2). However, the disaggregated results show contrasting patterns. In the decision-making component, France scores higher (6.75) than Spain (6.17). Conversely, Spain demonstrates significantly higher equity in both the management (7.33 versus 5.75) and distribution (7.37 versus 5.75) components (Fig. 2).

The results highlight notable differences in institutional performance between the two countries. As a result, considerations of equity may have differing impacts across the two countries, influencing the inclusiveness and perceived legitimacy of the decision-making process.

The French system appears significantly less clear in scope than the Spanish one. In Spain, despite a decentralized management structure, the roles and responsibilities of each actor are better defined, and information tends to flow more directly.

**Fig. 3:**
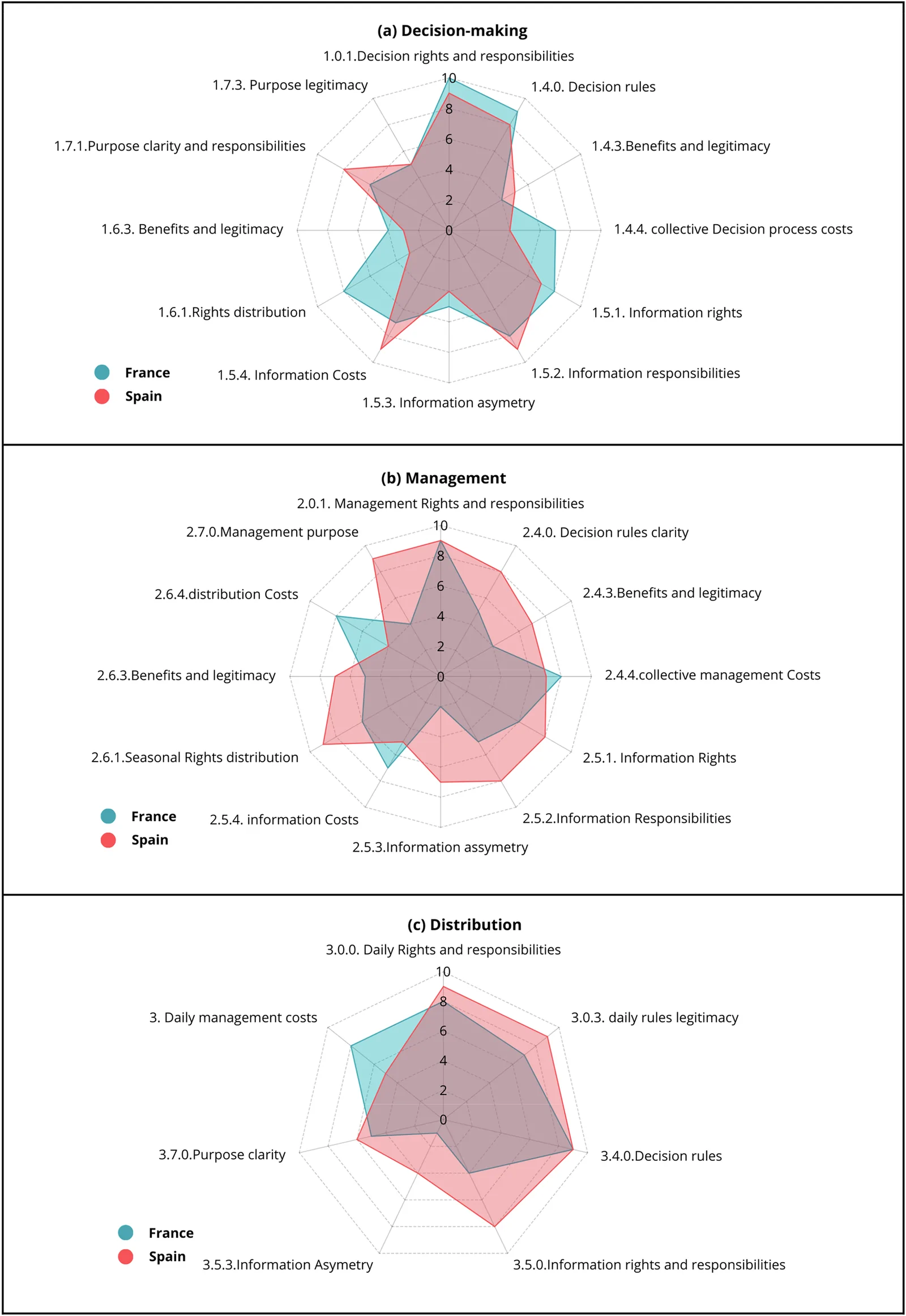
Results of the scoring system per components (Own realisation). offers an in-depth breakdown of the empirical findings, with sub-figures dedicated to each dimension of the management system. For each dimension, the results for France (blue) and Spain (red) are presented on the same plot in two different colors. **a** Represents the empirical results of applying the approach to the “Decision-making” dimension, comparing the French and Spanish hake fisheries in the Bay of Biscay. **b** Represents the empirical results of applying the approach to the “Management” dimension, comparing the French and Spanish hake fisheries in the Bay of Biscay. **c** Represents the empirical results of applying the approach to the “Distribution” dimension, comparing the French and Spanish hake fisheries in the Bay of Biscay.

The comparative analysis of Bay of Biscay Hake in France and Spain reveals contrasting institutional capacities and equity dynamics within their fisheries management systems. Differences are particularly evident in the decision-making component (Fig. 3), where Spain’s lower score highlights weaker institutional mechanisms to absorb transaction costs and ensure transparent criteria.

By contrast, France demonstrates stronger adaptive capacity, largely due to the role of producer Organizations. POs in France have a stronger entitlement to quota and thus a stronger legitimacy in making allocation decisions. Their local presence allows them to internalize transaction costs and tailor national regulation to local socio-economic contexts, resulting in a more flexible and context-sensitive form of equity. However, the absence of a unified national rationale for quota allocation across these organizations introduces inconsistencies and may weaken policy coherence. Spain’s more centralized allocation framework provides greater internal consistency and clarity in policy implementation. The lower entitlements held by Spanish POs place them in a weaker position with respect to allocation decisions.

Information and communication processes further differentiate the two systems. Spain’s decentralized political structure appears to enhance information dissemination and stakeholder outreach, leading to higher scores in the information-related dimension. In contrast, the French system benefits from a stronger institutional capacity to manage transaction costs but remains more fragmented in communication and coordination management levels.

Both countries operate within complex multi-level management arrangements involving EU, national, and regional authorities, as well as sectoral actors. These overlapping layers of responsibility can generate inefficiencies and information asymmetries, which, in turn, affect equity in access and participation.

These differences in management and allocation logic also have implications for equity, as they shape how access rights are justified, perceived, and negotiated among stakeholders.

## Discussion

Analyzing the theory of justice^41,52,53,63,64^ and the ways in which the scientific literature about fisheries management considers equity^25,65–67^ allowed us to frame a conceptual model of equity for fisheries management systems. From this framework, we developed an assessment approach allowing to assess the perceived equity of different fisheries management systems relating to the allocation of fishing possibilities.

A major challenge in evaluating fisheries management systems lies in the absence of a clear and operational definition of equity. This gap makes it difficult to identify the mechanisms that promote or hinder an equitable distribution of benefits and responsibilities among stakeholders. Examining the underlying social contract of a fisheries management system can provide valuable insights into where and how equity might be improved. By explicitly clarifying the roles, expectations, and power relations embedded in the system, such an approach can highlight structural inequities and inform more equitable policy design. Furthermore, the reduction of transaction costs may contribute to enhancing equity by ensuring more equitable access to resources and decision-making processes.

However, this empirical application has some limitations as an expert-based approach. The analysis may not capture the actual practices and informal dynamics among stakeholders. Extending the study directly to the stakeholders themselves, including fishers or representatives of co-management bodies from the administrations, would produce deeper outcomes. Their involvement would provide deeper insights into operational mechanisms, local perceptions, and context-specific challenges. Furthermore, such a participatory approach would improve understanding of information flows and potential asymmetries affecting real-time decision making.

While the contrasted Spanish and French fisheries management systems regulating access to the same fish stock provide a valuable case study within the EU, this duality also raises broader questions about how such systems compare to other models internationally (Territorial Use Rights for Fisheries (TURFs) in Chile, or ITQs in New Zealand and Iceland for example) where equity, legitimacy and adaptive governance are addressed through different institutional and cultural arrangements^2,68^.

This study raises several critical questions related to equity that warrant further investigation. First, how do informal networks and communication channels among stakeholders influence not only the decision-making process but also the equitable participation of diverse actors in multi-level management systems? Second, to what extent do information asymmetries impact fairness in compliance and responsiveness during rights distribution, and what mechanisms could be introduced to reduce these disparities? Third, how might differences in management structures between countries affect the equity in shared fisheries management? Fourth, what role do co-management institutions play in reducing transaction costs, and how might this contribute to enhancing equity? Finally, how can stakeholder participation be structured to effectively ensure equitable allocation of fishing possibilities within the system? The analysis also invites reflection on whether fisheries governance should aspire to “maximum equity” mirroring the pursuit of MSY in biological terms and MEY in economic terms, where ensuring that the distribution and legitimacy of fishing rights allocation is given equivalent standing to the objectives of ecological sustainability and economic efficiency. Addressing these questions is essential to deepen our understanding of equity in fisheries management.

To address the questions raised, it would also be necessary to extend the scope of the study. Future research should incorporate data collection through direct engagement with stakeholders to capture their perspectives, experiences and informal interactions. Comparative case studies across different management and social contexts could also illuminate how structural differences influence equity outcomes. Deeper research can validate and refine the approach, ultimately supporting a more equitable fisheries management system.

Understanding the social contract in fisheries management requires examining its cultural, social and institutional dimensions, as these shape the objectives, rules, and mechanisms of inclusion or exclusion within the fisheries system in a given society. Depending on the context, the fisheries social contract may have different priorities, thereby influencing which actors are legitimized to fish, monitor and decide, how decisions are made, and how rights are allocated. A compelling example of this dynamic is the use of preferential rights, which can serve as a mechanism to restore equity by addressing historical disparities or structural inequalities^69^. These preferential systems illustrate how social contracts are not merely static but adaptive tools that reflect local societal values and power dynamics, ultimately determining who benefits from fisheries management.

The concept of equity in fisheries management is not universal but rather culturally and contextually sensitive, taking on distinct forms depending on the social contracts, legal frameworks, and cultural values, whether rooted in collectivist traditions (e.g., Cuba^70^) or individualistic market principles (e.g., USA^71^, Iceland^2^), also explaining why there is research to date. By examining how social contracts shape the legitimacy of fishing rights allocation across diverse contexts, this analysis lays the ground for developing a unified methodological framework that could be adopted by intergovernmental institutions such as FAO to complement existing guidelines^72^. Such an approach would provide a standardized tool for evaluating how different systems address equity in fisheries management.

Moreover, the approach developed here could be applied beyond fisheries, offering valuable insights for the governance of other common-pool resources such as land and water.

## Methods

### Literature review

To inform the development of the equity framework and assessment approach, a review of the literature was conducted. The review focused on three complementary domains: theories of justice, fisheries management, and justice in ocean management. For fisheries management and justice in ocean governance, the focus was primarily on the most recent literature to capture current advancements and innovative practices. In contrast, for theories of justice, the analysis also included foundational and classical texts to understand the conceptual roots and philosophical underpinnings of equity. By integrating insights from these domains, we identified key principles and attributes relevant to equity in fisheries management, as well as gaps in existing approaches.

### Design of the approach

To refine and identify the assessment approach, a targeted review of the literature on common-pool resource management, the role of institutions, and transaction costs was conducted. Insights from these areas helped identify the structural and operational factors that influence equity in fisheries access rights allocation. Together, the conceptual review of justice and fisheries management and the targeted review of institutional and operational factors provide a comprehensive foundation for developing an equity assessment approach, bridging theoretical principles with practical implementation considerations.

### Case studies

To apply the assessment approach, each case study was assessed by the co-authors with the greatest relevant expertise in that fishery, who completed the full set of questions. The initial responses were then reviewed collectively by all co-authors, who proposed refinements. No substantive disagreements between co-authors arose during this process. Their raw responses have been reviewed and refined for clarity and relevance (Supplementary Tables 1 and 2).

## Supplementary information

Supplementary materials.docx (1).

## Data Availability

All data supporting the findings of this study are available within the paper and its Supplementary Information. Pilot approach's results from experts are provided in Supplementary Tables 1 and 2.
