# Supplementary material for: A practical approach to assess equity in fisheries management
Source: NPJ Ocean Sustain. 2026 Jun 3;5(1):45. doi: 10.1038/s44183-026-00210-4 (PMC13541608; doi:10.1038/s44183-026-00210-4)
Supplement: Supplementary file 1 [file 44183_2026_210_MOESM1_ESM.pdf]

Supplementary materials

Supplementary Table 1.

Supplementary table 1 - Empirical approach's results from an expert in the French case. (Own realisation)

Topics	French score	Rationale for the score
1.0.1. Decision rights and responsibilities	10	The CFP for Europe and the Code rural et de la pêche maritime in France provide detailed descriptions of rights and responsibilities.
1.4.0. Decision rules	9	The CFP's guidelines and French government mechanisms identify clear roles, responsibilities and processes.
1.4.3. Benefits and legitimacy	4	Despite the clarity of the regulatory framework, disparities in stakeholder representation persist, leading to conflictual views regarding the legitimacy and fairness of the decision-making process and allocation outcomes.
1.4.4. Collective decision process costs	7	CFP institutions and French decision arenas are well-established and actively engaged on a regular basis.
1.5.1. Information rights	8	National administration and industry representatives receive quota information resulting from European decisions (based on the relative stability principle defined by the CFP)
1.5.2. Information responsibilities	8	Quota information at the national level is shared with POs by the administration; POs are responsible for the dissemination of information to their individual members.
1.5.3. Information asymmetry	5	Despite the existence of structured consultation processes, certain actors may remain unaware of key consultations or information.
1.5.4. Information costs	7	Despite the role of Fisheries Committees and POs in facilitating the dissemination of information, some actors perceive a lack of mechanisms enabling information to be accessible to everyone
1.6.1. Rights distribution	8	The CFP's relative stability rule ensures a predictable distribution of fishing opportunities at the international level, while at the national level, quotas are allocated based on historical catch records. This is explicit in the fisheries law.
1.6.3. Benefits and legitimacy	4	Quota swaps between Member States are conducted routinely. Some states rely heavily on others to access specific quotas.
1.7.1. Purpose clarity and responsibilities	6	The CFP provides a formal framework of objectives and responsibilities balancing ecological, economic

		and social goals, which is not totally clear. The practice to achieve it remains unclear and undocumented.
1.7.3. Purpose legitimacy	5	Despite the CFP's formalisation of purpose, its implementation and decisions are not universally endorsed by all stakeholders.
2.0.1. Management rights and responsibilities	9	POs operate under clear internal rules and procedure, with variability in how quota shares are managed internally.
2.4.0. Decision rules clarity	5	While POs establish their own internal governance and decision-making processes, these may appear less transparent to external stakeholders.
2.4.3. Benefits and legitimacy	3	Within POs, decisions are made through internal voting mechanisms, where representatives hold decision-making authority.<br>Even if the PO membership is voluntary, its regional implementation does not totally allow fishers to move as they want. This may explain why even if some fishers are members, they may feel their interests are not well represented.
2.4.4. Collective management costs	8	POs and Fisheries committees support the administrative and monitoring costs
2.5.1. Information rights	6	Management involves multiple layers of authority, including POs, the national fisheries committee, Member States, and EU institutions.
2.5.2. Information responsibilities	5	While POs are responsible for disseminating information to their members, the specific types of information they are required to provide are not always clearly defined.
2.5.3. Information asymmetry	2	Information asymmetry exists among government bodies, POs and individual fishers, with certain stakeholders having privileged access to data, consultations, and decision-making processes.
2.5.4. Information costs	7	The administration and POs support the costs of data collection, research and management activities.
2.6.1. Seasonal rights distribution	6	On an annual basis, POs determine the internal rules managing quota allocation, practices and operational procedures.
2.6.3. Benefits and legitimacy	5	The annual distribution of quotas is largely managed by POs, which establish their own criteria and processes for allocating fishing rights. This allocation is considered unfair by some industry members.
2.6.4. Distribution costs	8	POs support costs for their members, including the quota management, monitoring and participation in decision-making processes.
2.7.0. Management purpose	4	In the absence of a general set of priorities at the national level, each PO defines its own management priorities. This is considered a limitation by some

		industry participants and other stakeholders (e.g.environmental NGOs)
3.0.0. Daily rights and responsibilities	8	POs exercise significant control over the consumption and reallocation possibilities during the fishing season.
3.0.3. Daily rules legitimacy	7	During the fishing season, POs hold considerable autonomy to reallocate quotas, although this authority is shared with fishing committees. However, the ultimate responsibility for closing fisheries rests with government bodies, prompting questions about the legitimacy of POs as management institutions.
3.4.0. Decision rules	9	Decisions regarding the closure of fisheries are clearly outlined in regulatory frameworks.
3.5.0. Information rights and responsibilities	4	Despite the existence of defined procedures, certain aspects of fisheries management such as control and monitoring procedures, remain ambiguous for some stakeholders for whom the information and the information provider remain unclear.
3.5.3. Information asymmetry	1	There is a significant disparity between POs and fishing committees, as well as other non-PO stakeholders, in terms of access to information, especially in terms of quota allocation and uptake.
3.7.0. Purpose clarity	5	The primary objective of the quota management system is to ensure that allocated quotas are not exceeded. This can be questioned by some stakeholders, who consider that other objectives should be taken into account (e.g. improving the economic efficiency of fishing, or reducing the environmental impacts of fishing)
3. Daily management costs	8	Information, control and enforcement are shared between POs and the administration.

Supplementary Table 2.

Supplementary table 2 - Empirical approach's results from an expert in the Spanish case. (Own realisation)

**Topics**	**Spanish score**	**Rationale for the score**
1.0.1. Decision rights and responsibilities	9	The regulatory framework for the Spanish hake fishery is clearly defined under the CFP and national legislation.
1.4.0. Decision rules	8	Clear and well-defined, but some stakeholders consider that the different community rules could undertake small contextual changes.

1.4.3. Benefits and legitimacy	5	Not all the stakeholders agree with the CFP rules. Discrepancies arise at the interface of the CFP's goals and local socio-economic realities.
1.4.4. Collective decision process costs	4	Although the CFP and Spanish institutions provide a structured framework, some stakeholders consider that operational mechanisms for adaptive management are insufficient, and not totally supported by the co-management institutions.
1.5.1. Information rights	7	While the CFP and Spanish laws are well-defined, their accessibility and comprehension vary significantly among stakeholders.
1.5.2. Information responsibilities	9	The CFP and Spanish regulatory framework clearly outlines who is responsible for disseminating information.
1.5.3. Information asymmetry	4	While access to information is theoretically equal, asymmetries persist in how stakeholders access, read and use this information.
1.5.4. Information costs	9	While the decentralised management system could limit the effectiveness of information transmission, the information chains are generally considered to work well.
1.6.1. Rights distribution	3	While the CFP's general rules are explicit, they have not been translated into more precise criteria by the Spanish administration.
1.6.3. Benefits and legitimacy	3	Not all stakeholders in the Spanish hake fishery perceive the current regulatory framework as legitimate.
1.7.1. Purpose clarity and responsibilities	8	The purpose of the CFP is generally considered to be clear.
1.7.3. Purpose legitimacy	5	While the CFP's framework is not universally validated by all the stakeholders, specific provisions like article 17 demonstrate effort to align regulations with industry needs, leading to mixed views.
2.0.1. Management rights and responsibilities	9	Responsibilities for fisheries management vary by region and fishing zone, but rules themselves are perceived to be clear within each context.
2.4.0. Decision rules clarity	8	POs and Cofradias each follow their own processes, shaped by regional practices, but these are generally considered to be clear.
2.4.3. Benefits and legitimacy	7	Co-management institutions in the hake fishery are well-organized and widely regarded as legitimate. But the legitimacy of the government decisions are questioned by some stakeholders.
2.4.4. Collective management costs	7	POs, Cofradias and local governments bear the seasonal cost of hake fishery management. However, small-scale operations face greater challenges due to limited resources.

2.5.1. Information rights	8	The information access for the hake fishery is clear. The diversity of actors reveal different understandings of who can access the information.
2.5.2. Information responsibilities	8	The entities responsible for the dissemination of information are clearly defined.
2.5.3. Information asymmetry	7	Decentralization in Spain facilitates information access among government bodies, POs, fishers and Cofradias supported by the Federation of Cofradias. While large-scale fishing holds more influence, asymmetries in information access are largely mitigated by this, although some asymmetries are perceived to remain.
2.5.4. Information costs	5	Co-management institutions support information management and availability. However the system is perceived to remain imperfect.
2.6.1. Seasonal rights distribution	9	State laws provide clarity for part of the hake fleet, particularly large-scale vessels. However, coastal fishers often find these rules less accessible.
2.6.3. Benefits and legitimacy	7	The national distribution of quotas is generally perceived as legitimate, though small-scale fishers may feel excluded from that process.
2.6.4. Distribution costs	4	There is a perception that the major costs are borne by the smallest operators, while these lack representation in the decision-making processes.
2.7.0. Management purpose	9	The transposition of CFP plans into Spanish hake fishery management is context-dependent, reflecting regional and local priorities.
3.0.0. Daily rights and responsibilities	9	Daily management of the Hake fishery by Cofradias and POs is well-defined and operational.
3.0.3. Daily rules legitimacy	9	Cofradias and POs are widely recognized as legitimate institutions in the hake fishery, largely due to their deep roots in local fishing communities.
3.4.0. Decision rules	9	National laws and the internal rules of Cofradias and POs provide clear and consistent guidelines.
3.5.0. Information rights and responsibilities	8	The information access and dissemination responsibilities are well-defined between POs and Cofradias.
3.5.3. Information asymmetry	4	At the local level, limited time and capacity among fishers and Cofradias can hinder their ability to fully master and/or engage with complex regulatory information.
3.7.0. Purpose clarity	6	The management purpose is not always considered to be clear at the level of local application. Some stakeholders consider that the application of article 17 of the CFP is incomplete.
3. Daily management costs	5	Information is readily available to stakeholders, but mechanisms to cover associated costs are considered to be lacking. The system is considered imperfect, particularly for resource-limited actors.

Supplementary Table 3.

Supplementary table 3 - Ten-level scoring scale. (Own realisation)

Component	Question	Scale explanation
Decision-making	Are the roles of each stakeholder in the decision process explicit and clear?	1 = No definition of the roles in the decision-making process
		10 = Perfectly clear and complete definition of the roles in the decision-making process
	Is the pluri-annual decision-making process clearly defined?	1 = No definition of how long-term decisions are made
		10 = Perfectly clear and complete definition of how long-term decisions are made
	Is the long-term decision process legitimate?	1 = The long-term decision-making process is illegitimate
		10 = The long-term decision-making process is fully legitimate
	Are there institutions or mechanisms in place to support the decision-making process (help with access to information, public participation, discussion/debate forums)?	1 = No mechanisms or institutions supporting the decision-making process
		10 = Existing mechanisms or institutions supporting the decision-making process
	Is the pluri-annual information access clearly defined?	1 = No definition of who can access the information
		10 = Perfectly clear definition of who can access the information
	Is it clearly defined who is responsible to give access to the information?	1 = No definition of who is responsible to give access to the information
		10 = Perfectly clear and complete definition of who is responsible to give access to the information
	Is the pluri-annual information equally accessible for all the stakeholders?	1 = The stakeholders don't get the same access to the information

		10 = The stakeholders receive the same access to the information
	Are there any institutions or mechanisms in place to support the distribution of clear information?	1 = No mechanisms or institutions supporting the distribution and reading of clear information
		10 = There are institutions and mechanisms supporting the distribution and reading of clear information
	Are the pluri-annual rules to distribute the fishing possibilities clearly defined?	1 = No definition of the pluri-annual rules to distribute fishing rights
		10 = Perfectly clear and complete definition of the pluri-annual rules to distribute fishing rights
	Are the pluri-annual distribution rules of fishing possibilities legitimate?	1 = The pluri-annual distribution rules of fishing rights is illegitimate
		10 = The pluri-annual distribution rules of fishing rights is fully legitimate
	Is the purpose of the pluri-annual fishery management clearly defined?	1 = No definition of the purpose of the long-term fishery management
		10 = Perfectly clear and complete definition of the purpose of the long-term fishery management
	Is the purpose of the fisheries policies legitimate?	1 = The general purpose of the fishery policy is not legitimate
		10 = The purpose of the fishery policy is fully legitimate
Management	Are the roles of each stakeholder in the seasonal fisheries management explicit and clear?	1 = No definition of the roles in the seasonal decision-making process
		10 = Perfectly clear and complete definition of the roles in the seasonal decision-making process
	Is the process to decide the seasonal management strategy clearly defined?	1 = No definition of how seasonal decisions are made
		10 = Perfectly clear and complete definition of how seasonal decisions are made
	Is the way of deciding the seasonal management strategy legitimate ?	1 = The seasonal decision-making process is illegitimate

		10 = The seasonal decision-making process is fully legitimate
	Are there institutions or mechanisms in place to support the seasonal decision-making process?	1 = No mechanisms or institutions supporting the decision-making process
		10 = Existing mechanisms or institutions supporting the decision-making process
	Is it clear who gets the right to access the information at the beginning of the season to edit the fisheries management strategy?	1 = No definition of who can access the information
		10 = Perfectly clear definition of who can access the information
	Is it clearly defined who is responsible to provide the information at the beginning of the season to be able to take the seasonal decision?	1 = No definition of who is responsible to give access to the information
		10 = Perfectly clear and complete definition of who is responsible to give access to the information
	Is the information equally accessible for all the stakeholders?	1 = The stakeholders don't get the same access to the information
		10 = The stakeholders receive the same access to the information
	Are there any institutions or mechanisms in place to support the distribution of clear information?	1 = No mechanisms or institutions supporting the distribution and reading of a clear information
		10 = There are institutions and mechanisms supporting the distribution and reading of a clear information
	Is it clearly defined who receives the fishing rights in the seasonal fisheries management strategy?	1 = No definition of the seasonal rules to distribute fishing rights
		10 = Perfectly clear and complete definition of the seasonal rules to distribute fishing rights
	Are the annual distribution rules of fishing possibilities legitimate?	1 = The seasonal distribution rules of fishing rights is illegitimate
		10 = The seasonal distribution rules of fishing rights is fully legitimate

	Are the costs associated with the seasonal distribution of the fishing rights supportable?	1 = No mechanisms or institutions supporting the seasonal distribution of fishing rights
		10 = Existing institutions and mechanisms supporting the seasonal distribution of fishing rights
	Is the purpose of the seasonal management strategy and the actors responsible for it clearly defined?	1 = No definition of the purpose of the seasonal fishery management
		10 = Perfectly clear and complete definition of the purpose of the seasonal fishery management
Distribution	Are the rights and responsibilities well defined in the infra-annual fisheries management system?	1 = No definition of the roles and responsibilities in the daily management
		10 = Perfectly clear and complete definition of the roles and responsibilities in the daily management
	Is the previous repartition of the daily rights and responsibilities between stakeholders legitimate?	1 = The repartition of daily rights and responsibilities is illegitimate
		10 = The repartition of daily rights and responsibilities is fully legitimate
	Does it clearly define how decisions are made during the fishing season?	1 = No definition of the daily decision-making process
		10 = Perfectly clear and complete definition of the daily decision-making process
	Is it clearly defined who gets the rights to access the infra-annual information to manage the fishery ? and who is responsible to give this access?	1 = There is no definition of who can access the daily fishery information and who is responsible for granting access to this information.
		10 = Perfectly clear and complete definition of who can access the daily fishery information and who is responsible for granting access to this information.
	Is daily information accessible in the same way to all stakeholders?	1 = The stakeholders don’t get the same access to the daily information
		10 = The daily information is equally accessible to all stakeholders
	Is the purpose of the infra-annual management clear?	1 = No definition of the purpose of the infra-annual fishery management

		10 = Perfectly clear and complete definition of the purpose of the infra-annual fishery management
	Are there institutions and mechanisms supporting the intra-annual management of the fishery?	1 = No mechanisms or institutions supporting the daily management of the fishery
		10 = Existing mechanisms or institutions supporting the daily management of the fishery
